# Long-term trends in the burden of inflammatory bowel disease in China over three decades: A joinpoint regression and age-period-cohort analysis based on GBD 2019

**DOI:** 10.3389/fpubh.2022.994619

**Published:** 2022-09-07

**Authors:** Yang Zhang, Jiali Liu, Xiao Han, Hui Jiang, Liming Zhang, Juncong Hu, Lei Shi, Junxiang Li

**Affiliations:** ^1^Department of Gastroenterology, Dong Fang Hospital, Beijing University of Chinese Medicine, Beijing, China; ^2^Graduate School, Beijing University of Chinese Medicine, Beijing, China; ^3^Department of Gastroenterology, Xiyuan Hospital of China Academy of Chinese Medical Sciences, Beijing, China

**Keywords:** inflammatory bowel disease, disease burden, joinpoint regression, age-period-cohort analysis, epidemiological study

## Abstract

**Background:**

To study the corresponding strategies to control inflammatory bowel disease (IBD), a comprehensive assessment of the disease burden is required. Herein, we present long-term trends in the burden of IBD in China over the last three decades, as well as its epidemiological features.

**Methods:**

We characterized the burden of IBD in China using the GBD 2019 methods and results, based on prevalence, incidence, mortality, years lost (YLLs), years lived with disability (YLDs), and disability-adjusted life years (DALYs) estimated using the DisMod-MR 2-1. We also used joinpoint and age-period-cohort (apc) analysis methods to interpret IBD epidemiological characteristics and compare them to global prevalence trends.

**Results:**

The age-standardized incidence and mortality rates in both sexes changed from 1.47 (95% CI: 1.24, 1.74) to 3.01 (95% CI: 2.59, 3.50) and from 0.86 (95% CI: 0.59, 1.16) to 0.30 (95% CI: 0.24, 0.35) per 100,000 people in China from 1990 to 2019. The age-standardized DALY rate in China decreased from 24.47 (95% CI: 17.88, 30.19) per 100,000 people in 1990 to 13.10 (95% CI: 10.29, 16.31) per 100,000 people in 2019. The average annual percentage change (AAPC) in age-standardized incidence, prevalence, and mortality rates for IBD in China were 2.51 (95% CI: 2.44, 2.57), 2.53 (95% CI: 2.41, 2.66), and −3.62 (95% CI: −3.85, −3.39). The effects of age, period, and cohort on incidence and mortality rates differed.

**Conclusions:**

The increasing age-standardized prevalence rates are contributed to by the reduction in age-standardized mortality rates and DALYs, compounded by the rise in the age-standardized incidence rates between 1990 and 2019 in China. The burden of IBD in China will be a major public health challenge, given the country's large population base and aging population.

## Introduction

Inflammatory bowel disease (IBD) is a chronic systemic inflammatory illness with two subtypes, ulcerative colitis (UC) and Crohn's disease (CD) (1). UC affects only the colon whereas CD affects the entire gastrointestinal tract, from mouth to anus, with the formation of strictures, abscesses, or fistulas that invade surrounding organs or the perianal skin (2, 3). These disorders primarily affect the gastrointestinal tract; however, extraintestinal symptoms can impact numerous organ systems (4). The initial clinical presentation of the disease is determined by the amount and activity of the disease and may include stomach pain, diarrhea with blood and mucus passing, fever, clinical symptoms of bowel blockage, anemia, and raised levels of test markers of inflammation (5). IBD has no cure for now (2). Long-term chronic inflammation raises the risk of additional malignant processes that necessitate lifelong care to prevent or delay progression (6).

IBD was previously more common in Western high-income countries and infrequently reported in Asia, Africa, and Latin America (7). In the twenty-first century, the epidemiological paradigm has evolved, with rates stabilizing in Western countries and rapidly growing in newly industrialized countries in South America, Eastern Europe, Asia, and Africa (8, 9). IBD has spread throughout the world. In China, the incidence of IBD is increasing along with economic development and lifestyle changes (10). The increased prevalence of IBD will result in a major increase in the disease burden due to the negative impact on quality of life and the high expenditure associated with its protracted course.

China is the world's most populous country, and the growing burden of IBD has piqued the medical community's interest (11, 12). However, the national IBD registry is still inadequate, and epidemiological reports are limited. The Global Burden of Diseases, Injuries, and Risk Factors Study of 2019 (GBD 2019) includes health statistics from over 200 nations worldwide, and researchers iterate on the most recent data and research techniques, with estimates updated throughout the time series (13, 14). The GBD 2019 results presently supersede those of prior GBD rounds. Based on the most recent GBD 2019 data, we present long-term trends in the burden of IBD in China over the past 30 years, including prevalence, incidence, mortality, years of life lived with disability (YLDs), years of life lost (YLLs), and disability-adjusted life years (DALYs).

## Methods

### Overview

The GBD 2019 report comprises 369 diseases and injuries and 87 risk factors in 204 countries and regions (15, 16), which includes estimations of numerous different models for disease and injury outcomes. The Cause of Death Ensemble model (CODEm), spatiotemporal Gaussian process regression (ST-GPR), and Bayesian meta-regression tool DisMod-MR were the main methods of estimating the prevalence, incidence, deaths, YLLs, YLDs, and DALYs by cause, age, sex, year, and location for the GBD 2019 (15).

Data resources, definitions, statistical modeling, and initiatives to improve data quality have all been previously described in detail (14, 17, 18). The Global Health Data Exchange GBD Results Tool (https://vizhub.healthdata.org/gbd-results/) was used to collect data on inflammatory bowel disease burdens in China from 1990 to 2019. All age-standardized rates, age-specific rates, including 95% uncertainty interval data, are available from GBD.

The Dongfang Hospital Beijing University of Chinese Medicine's Institutional Review Board ruled that this study did not require clearance because it used publicly available data.

### Joinpoint regression analysis

The joinpoint regression model is a collection of linear statistical models that were used to evaluate the trends in disease burdens attributable to IBD across time. This model's calculating approach is to estimate the changing rule of illness rates using the least square method, avoiding the non-objectivity of typical trend analyses based on linear trends. Calculating the square sum of the residual error between the estimated and actual values yields the turning point of the shifting trend. Joinpoint (version 4.9.1.0; National Cancer Institute, Rockville, MD, USA) was used to create this model. We also calculated the average annual percentage change (AAPC) and investigated if the fluctuation trend in different parts was statistically significant by comparing the AAPC to 0. A statistically significant *P*-value was less than 0.05.

### Age-period-cohort analysis

Age-period-cohort (apc) models are commonly used in sociology and epidemiology. Based on Poisson distributions, apc models can reflect temporal trends in incidence or mortality by age, period, and cohort. However, due to the linear relationship between age, period, and cohort, which makes estimating a unique set of effects for each age, period, and cohort difficult, the problem of non-identifiability may still exist (19). Researchers have attempted to address this problem from various perspectives, proposing various solutions such as intrinsic estimators (20), penalty function methods (21), estimation functions (22), and others; however, they still have some limitations. B. Carstensen thoroughly explains a technique for apc model analyses from the Lexis diagram (23, 24). This method was used for the apc analysis in this study. The GBD was queried for the incidence and mortality per 5-year age group from 1990 to 2019, as well as population estimates for each year (https://ghdx.healthdata.org/record/ihme-data/global-population-forecasts-2017-2100). GBD classified people under 5 years and over 95 years into one group, and for the purpose of apc model fitting, the age groups were defined as 0–4, 5–9, 10–14...95–100, with 0 in the figure indicating the under-five group. For a 5-year period (1990–1994, 1995–1999…2014–2019), the total number of cases of incidence or death, as well as the cumulative incidence and mortality rates for various age groups were calculated. We performed apc model fitting using the Epi package (version 2.46) in R (version 4.2.0 http://www.r-project.org). Residual deviations between models and AIC were compared to determine the optimal model.

## Results

### Descriptive analysis

In China, there were 51,500 (95% CI: 43900, 60500) new cases in 2019 and 4676 (95% CI: 3774, 5461) deaths due to IBD. Age-standardized rates in terms of prevalence (ASPR), incidence (ASIR), mortality (ASMR), DALYs, YLDs, and YLLs of IBD in 2019 were 47.06 cases (95% CI: 40.05, 54.99) per 100,000, 3.01 new cases (95% CI: 2.59, 3.5) per 100,000, 0.30 deaths (95% CI: 0.24, 0.35) per 100,000, 13.1 DALYs (95% CI: 10.29, 16.31) per 100,000, 7.07 YLDs (95% CI: 4.65, 9.86) per 100,000, and 6.02 YLLs (95% CI: 4.78, 6.95) per 100,000 (Table 1). The all-age numbers and age-standardized rates for males and females are presented in Table 1. It is clear that men have a higher disease burden than women (Table 1).

**Table 1:** All-age cases and age-standardized prevalence, incidence, deaths, YLLs, YLDs, and DALYs rates in 2019 for IBD in China.

**Measure**	**All-ages cases**			**Age-standardized rates per 100 000 people**		
	**Total**	**Male**	**Female**	**Total**	**Male**	**Female**
Prevalence	911045.10 (776346.59, 1069532.93)	484362.37 (410505.74, 571357.81)	426682.74 (365758.42, 497906.28)	47.06 (40.05, 54.99)	50.01 (42.46, 58.51)	44.28 (37.86, 51.39)
Incidence	51461.96 (43932.97, 60474.47)	28887.06 (24637.78, 33948.45)	22574.90 (19411.75, 26625.57)	3.01 (2.59, 3.50)	3.35 (2.88, 3.88)	2.65 (2.29, 3.08)
Deaths	4675.97 (3774.48, 5461.44)	2540.57 (1884.95, 3148.11)	2135.40 (1647.18, 2624.96)	0.30 (0.24, 0.35)	0.38 (0.30, 0.47)	0.24 (0.19, 0.30)
DALYs	232463.85 (179902.70, 291090.42)	128402.38 (98506.89, 161625.79)	104061.47 (79721.46, 134433.08)	13.10 (10.29, 16.31)	14.88 (11.53, 18.45)	11.56 (8.99, 14.78)
YLDs	135906.35 (89067.21, 191529.02)	71231.44 (47178.43, 100328.63)	64674.91 (42508.97, 90777.19)	7.07 (4.65, 9.86)	7.38 (4.87, 10.35)	6.79 (4.47, 9.48)
YLLs	96557.50 (75916.72, 112804.32)	57170.94 (41100.94, 70806.92)	39386.56 (30100.66, 48315.58)	6.02 (4.78, 6.95)	7.51 (5.66, 9.14)	4.77 (3.67, 5.80)

DALYs, disability-adjusted life-years; YLDs, years lived with disability; YLLs, years of life lost.

**Figure 1:**
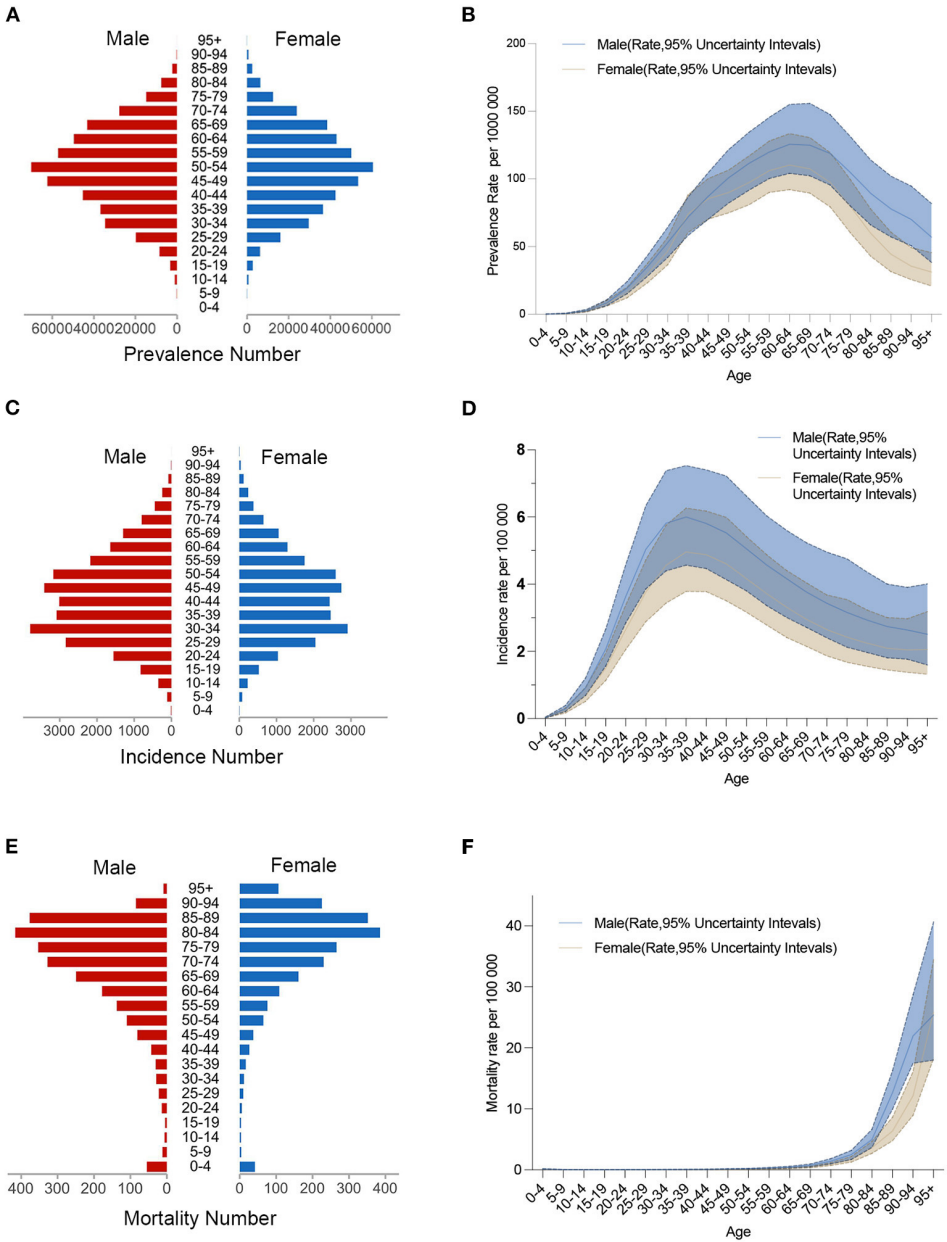
Age-specific numbers and age-standardized prevalence, incidence, and mortality rates of IBD in China, 2019. **(A)** Age-specific prevalence number. **(B)** Age-standardized prevalence rate. **(C)** Age-specific incidence number. **(D)** Age-standardized incidence rate. **(E)** Age-specific mortality number. **(F)** Age-standardized mortality rate.

Figure 1 shows the prevalence, incidence, mortality numbers (A, C, E), and age-standardized rates (B, D, F) of IBD for the different age groups in 2019. IBD is more prevalent in people over the age of 35, and it increases quickly between the ages of 30 and 69. Males are most affected between the ages of 65 and 69 and females between the ages of 60 and 64. In terms of incidence, similar tendencies are observed, with a substantial increase in incidence occurring after the age of 30. The highest incidence peaks occurred between the ages of 30 and 39. After the age of 65, the mortality rate increased dramatically. Surprisingly, men had higher prevalence, incidence, and mortality rates than women. Age-standardized DALYs, YLDs, and YLLs rates showed similar trends by sex and age group (Supplementary Figure 1).

**Figure 2:**
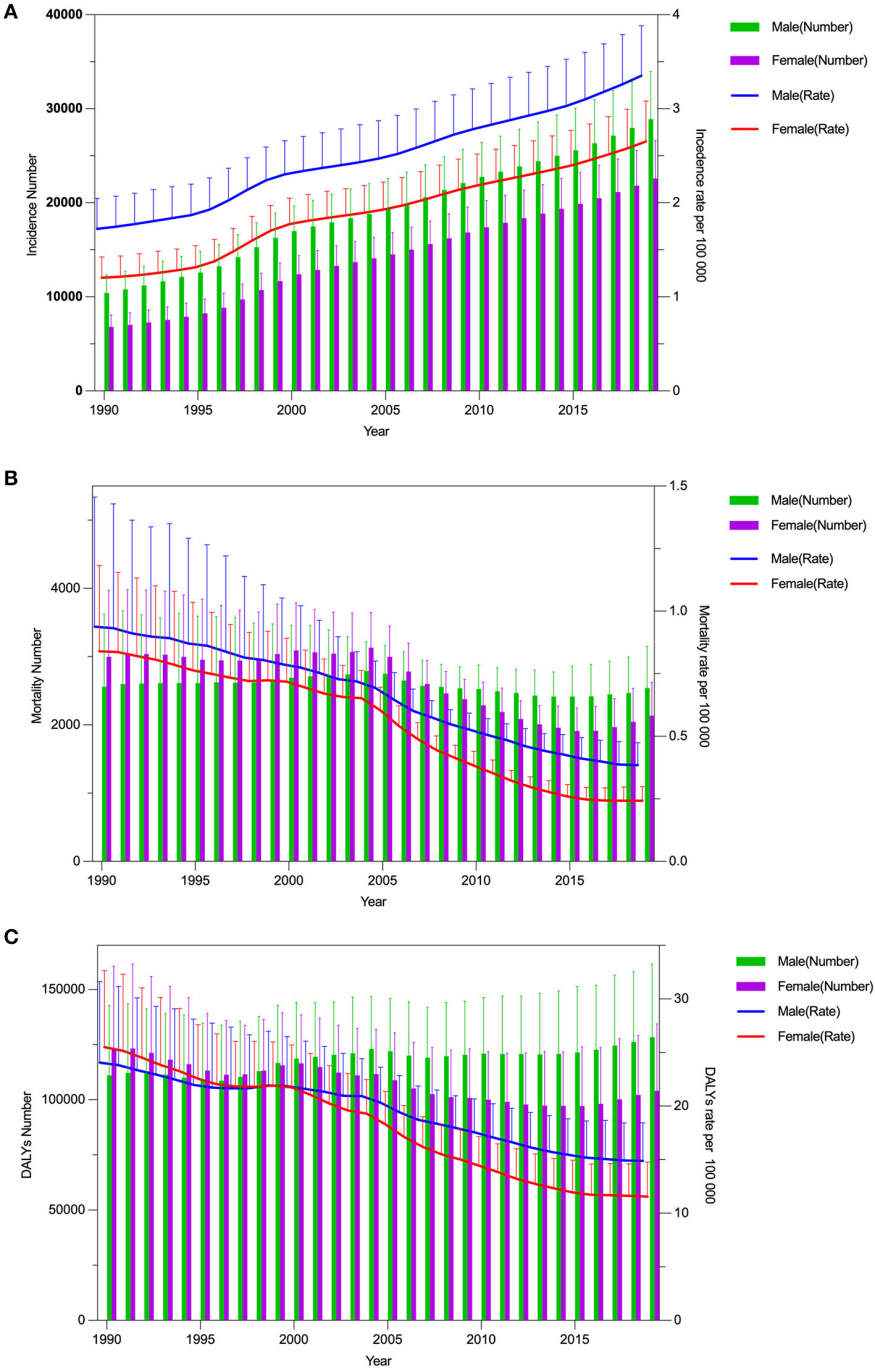
Trends in the all-age cases and age-standardized incidence, mortality, and DALYs rates of IBD by sex from 1990 to 2019. **(A)** Incidence number and rate. **(B)** Mortality number and rate. **(C)** DALYs number and rate.

The age-standardized incidence and mortality rates in both sexes changed from 1.47 (95% CI: 1.24, 1.74) to 3.01 (95% CI: 2.59, 3.50) and from 0.86 (95% CI: 0.59, 1.16) to 0.30 (95% CI: 0.24, 0.35) per 100,000 people in China from 1990 to 2019 (Supplementary Table 1). The age-standardized DALY in China decreased from 24.47 (95% CI: 17.88, 30.19) per 100,000 people in 1990 to 13.10 (95% CI: 10.29, 16.31) per 100,000 people in 2019 (Supplementary Table 1). When compared to global data, China's IBD burden has changed significantly. Figure 2 depicts the trends in the sex-specific all-age number and age-standardized rates of IBD incidence and mortality in China from 1990 to 2019. The sex-specific, age-standardized incidence and mortality rates for IBD fluctuated by calendar year. The disease incidence is generally increasing (Figure 2A) while mortality is gradually declining (Figure 2B). Both the male and female age-standardized DALYs are decreasing. However, it seems there is no decrease in the number of DALYs for men (Figure 2C). From 1990 to 2019, the sex-specific all-age number and age-standardized rates of IBD prevalence and YLDs in China increased, while the YLL burden decreased (Supplementary Figures 2A–C).

### Joinpoint regression analysis

**Figure 3:**
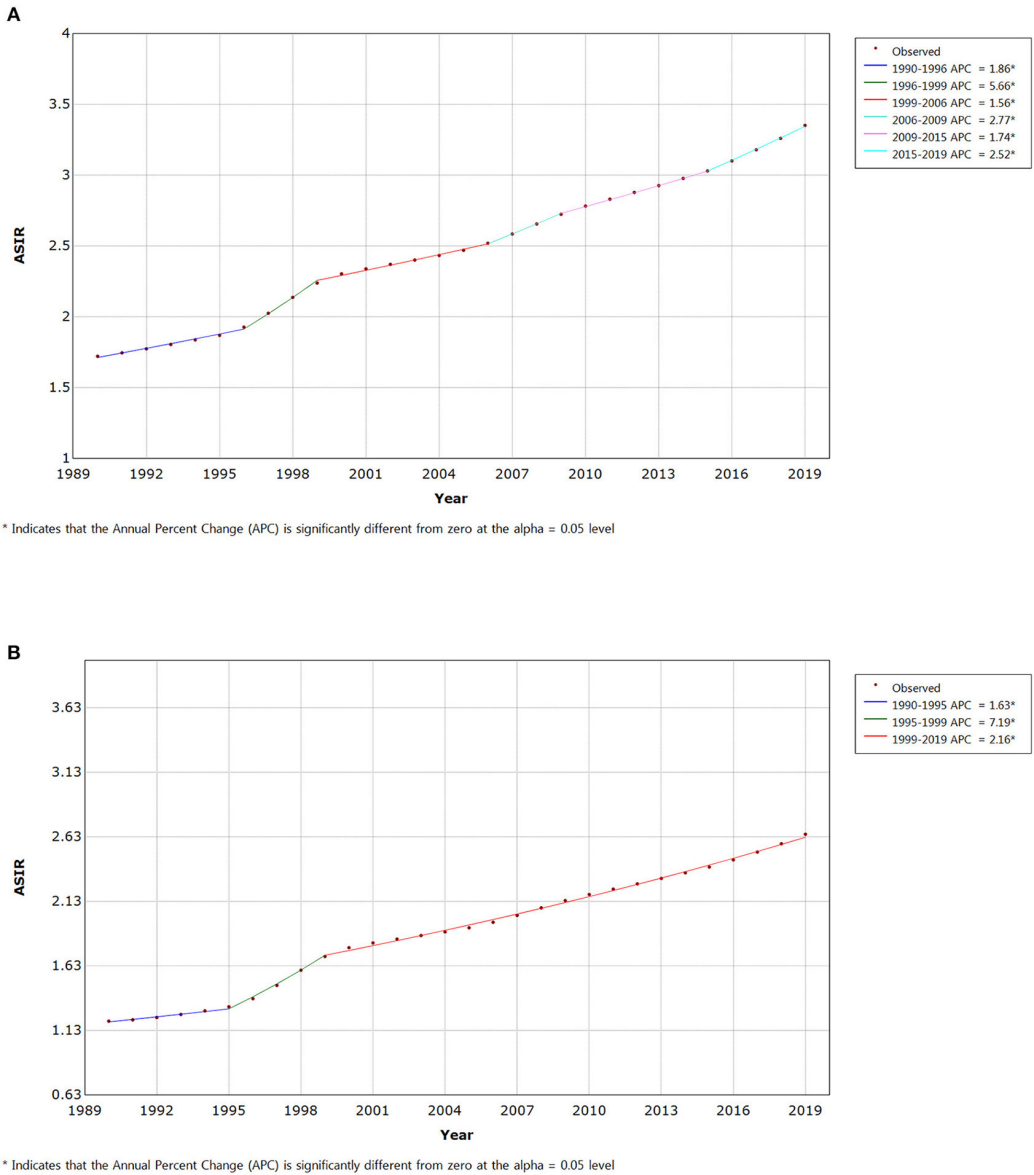
Joinpoint regression analysis of the sex-specific age-standardized incidence rate for IBD in China from 1990 to 2019. **(A)** Age-standardized incidence rate for males. **(B)** Age-standardized incidence rate for females.

**Figure 4:**
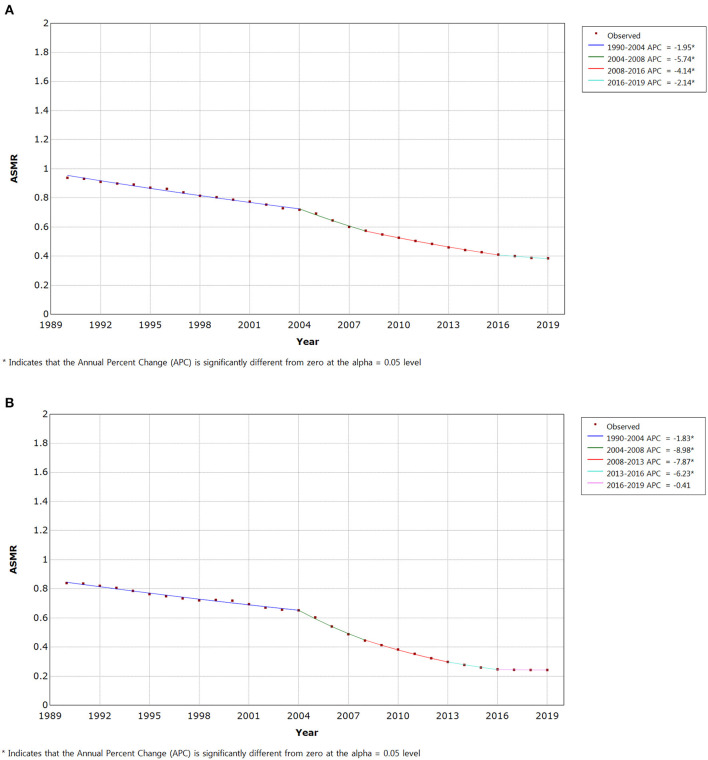
Joinpoint regression analysis of the sex-specific age-standardized mortality rate for IBD in China from 1990 to 2019. **(A)** Age-standardized mortality rate for males. **(B)** Age-standardized mortality rate for females.

Joinpoint regression analyses of the age-standardized incidence rates for IBD in China from 1990 to 2019 are shown in Figure 3. We found the disease incidence trend to significantly increase from 1995 to 1999 in both male (APC = + 5.66 (1996–1999), 95% CI: 5.37, 5.96) (Figure 3A) and female (APC = + 7.19 (1995–1999), 95% CI: 6.08, 8.30) populations (Figure 3B). Since 1999, the incidence trend has moderated in both sexes. However, it is still rising year on year, and the prevalence is on a similar upward trend (Supplementary Figure 3). The age-standardized mortality rate significantly decreased from 2004 to 2016 in both males (2004–2008 APC = −5.74, 2008–2016 APC = −4.14) and females (2004–2008 APC = −8.98, 2008–2013 APC = −7.87, 2013–2016 APC = −6.23) (Figures 4A,B). Joinpoint regression analyses of the age-standardized prevalence, incidence, and mortality rates in both sexes are shown in Supplementary Figure 4.

Table 2 shows the AAPCs in IBD incidence, prevalence, and mortality rates over three decades. Age-standardized incidence, prevalence, and mortality rates for IBD in China increased by 2.51 (95% CI: 2.44, 2.57), 2.53 (95% CI: 2.41, 2.66), and −3.62 (95% CI: −3.85,-3.39), respectively, from 1990 to 2019. Surprisingly, males had a lower AAPC of incidence, prevalence, and mortality rates than females (Table 2).

**Table 2:** Joinpoint regression analysis: trends in age-standardized incidence, prevalence, mortality rates (per 100,000 persons) among both sexes, males, and females in China, 1990–2019.

**Gender**	**ASIR**			**ASPR**			**ASMR**		
	**Period**	**APC (95% CI)**	**AAPC (95% CI)**	**period**	**APC (95% CI)**	**AAPC (95% CI)**	**period**	**APC (95% CI)**	**AAPC (95% CI)**
Both	1990–1995	1.69 (1.55, 1.82)	2.51 (2.44, 2.57)	1990–1993	0.12 (−0.32, 0.56)	2.53 (2.41, 2.66)	1990–2004	−1.72 (−1.84, −1.61)	−3.62 (−3.85, −3.39)
	1995–2000	5.26 (5.08, 5.44)		1993–1996	2.95 (2.07, 3.85)		2004–2007	−7.83 (−9.53, −6.09)	
	2000–2005	1.31 (1.14, 1.47)		1996–1999	11.87 (11.00, 12.75)		2007–2013	−6.36 (−6.64, −6.07)	
	2005–2010	2.54 (2.37, 2.71)		1999–2009	1.83 (1.76, 1.90)		2013–2016	−5.06 (−6.19, −3.91)	
	2010–2015	1.73 (1.56, 1.90)		2009–2019	1.17 (1.11, 1.23)		2016–2019	−1.04 (−1.77, −0.31)	
	2015–2019	2.57 (2.40, 2.75)							
Male	1990–1996	1.86 (1.81, 1.91)	2.33 (2.29, 2.38)	1990–1993	0.29 (−0.70, 1.29)	2.42 (2.10, 2.75)	1990–2004	−1.95 (−2.08, −1.82)	−3.11 (−3.26, −2.95)
	1996–1999	5.66 (5.37, 5.96)		1993–1996	2.93 (0.94, 4.96)		2004–2008	−5.74 (−6.57, −4.91)	
	1999–2006	1.56 (1.51, 1.60)		1996–1999	10.86 (8.96, 12.80)		2008–2016	−4.14 (−4.32, −3.96)	
	2006–2009	2.77 (2.49, 3.04)		1999–2002	2.63 (0.88, 4.41)		2016–2019	−2.14 (−2.96, −1.31)	
	2009–2015	1.74 (1.68, 1.80)		2002–2019	1.25 (1.19, 1.32)				
	2015–2019	2.52 (2.43, 2.60)							
Female	1990–1995	1.63 (1.13, 2.14)	2.75 (2.58, 2.91)	1990–1995	0.46 (0.18, 0.74)	2.71 (2.56, 2.85)	1990–2004	−1.83 (−1.97, −1.68)	−4.22 (−4.46, −3.97)
	1995–1999	7.19 (6.08, 8.30)		1995–1999	10.60 (9.95, 11.25)		2004–2008	−8.98 (−10.05, −7.90)	
	1999–2019	2.16 (2.11, 2.22)		1999–2002	2.98 (1.86, 4.12)		2008–2013	−7.87 (−8.35, −7.39)	
				2002–2011	1.83 (1.71, 1.95)		2013–2016	−6.23 (−7.71, −4.72)	
				2011–2019	1.22 (1.10, 1.34)		2016–2019	−0.41 (−1.33, 0.53)	

AAPC, average annual percent change presented for full period; APC, annual percent change; CI, confidence interval.

### The effects of age, period, and cohort on incidence and mortality rates

**Figure 5:**
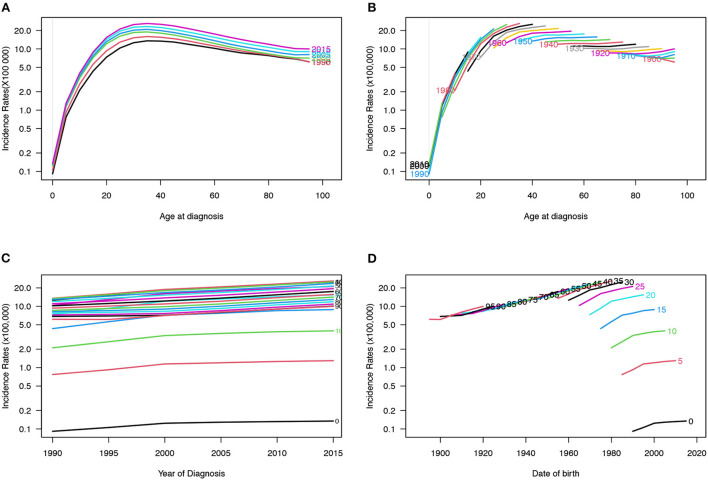
Incidence rates of IBD in China. **(A)** The age-specific incidences rates of IBD according to time periods; each line connects the age-specific incidence for a 5-year period. **(B)** The age-specific incidences rates of IBD according to birth cohort; each line connects the age-specific incidence for a 5-year cohort. **(C)** The period-specific incidence rates of IBD according to age groups; each line connects the birth cohort-specific incidence for a 5-year age group. **(D)** The birth cohort-specific incidence rates of IBD according to age groups; each line connects the birth cohort-specific incidence for a 5-year age group.

**Figure 6:**
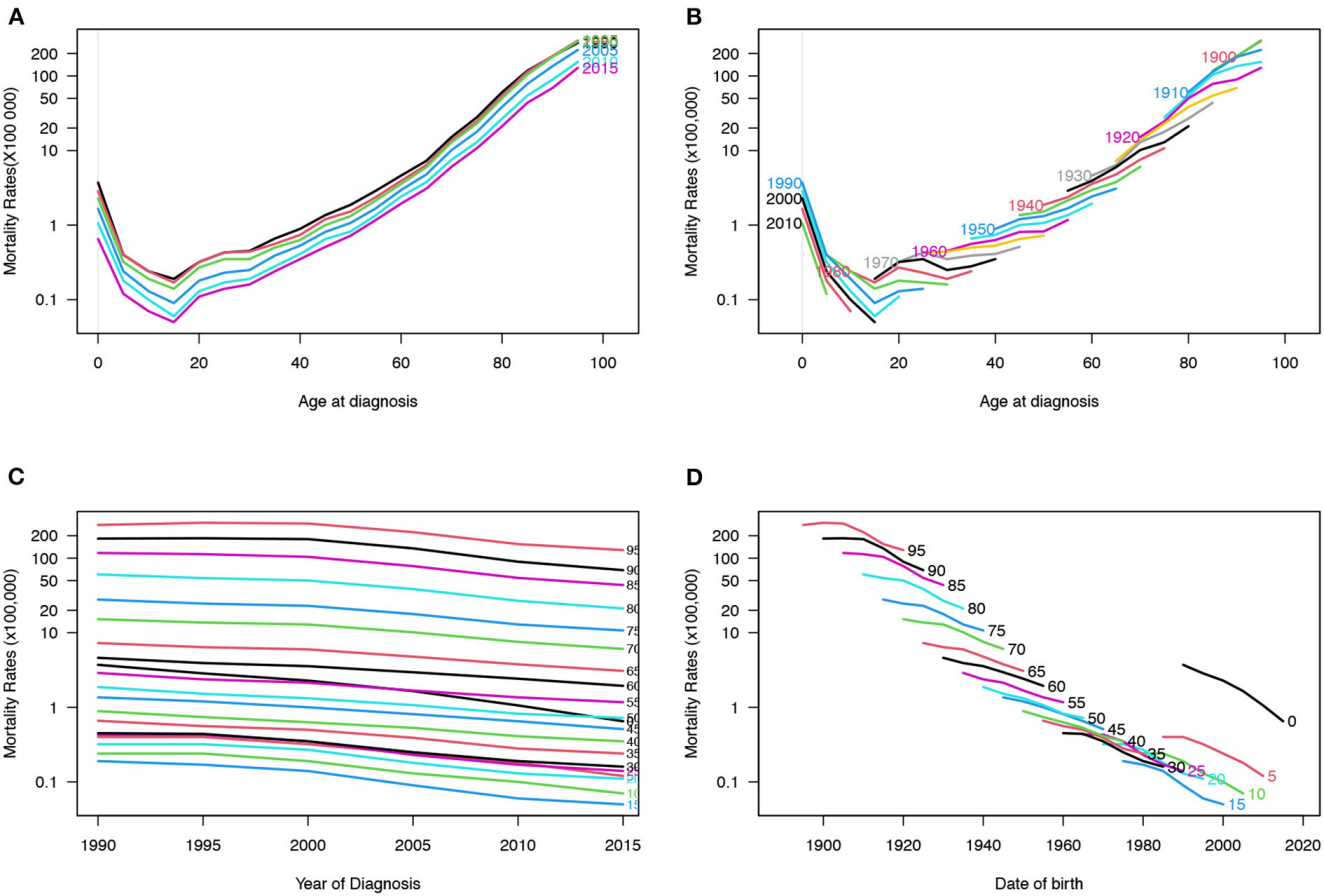
Mortality rates of IBD in China. **(A)** The age-specific mortality rates of IBD according to time periods; each line connects the age-specific mortality for a 5-year period. **(B)** The age-specific mortality rates of IBD according to birth cohorts; each line connects the age-specific mortality for a 5-year cohort. **(C)** The period-specific mortality rates of IBD according to age group; each line connects the birth cohort-specific mortality for a 5-year age group. **(D)** The birth cohort-specific mortality rates of IBD according to age groups; each line connects the birth cohort-specific mortality for a 5-year age group.

Figures 5A, 6A depict the incidence and mortality trends by age for the 1990, 1995, 2000, 2005, 2010, and 2015 periods. The incidence rate increased rapidly between the ages of 0 and 40 while the incidence rate after 40 showed a slowly-decreasing trend. However, after 20 years of age, the mortality rate increased rapidly with age. Figures 5B, 6B depict the cohort trends in IBD incidence and mortality for various age groups. Figures 5C, 6C depict the trends in IBD incidence and mortality rates for various age groups between 1990 and 2019. The incidence rate increased nearly over time for all age groups while the mortality rate decreased slightly. The incidence rate is higher in young people while the mortality rate is higher in old people. Figures 5D, 6D depict changes in incidence and mortality rates based on cohorts for specific age groups. The incidence of IBD increased with age; however, there were no significant differences after the age of 40. Mortality rates continued to rise with each passing year of birth, with the 10–15 age group having the lowest rate. Surprisingly, the mortality rate in the 0–4 age group was also higher. The variations of age, period, and cohort on incidence and mortality rates among males and females are shown in the Supplementary Figures 5–8.

**Figure 7:**
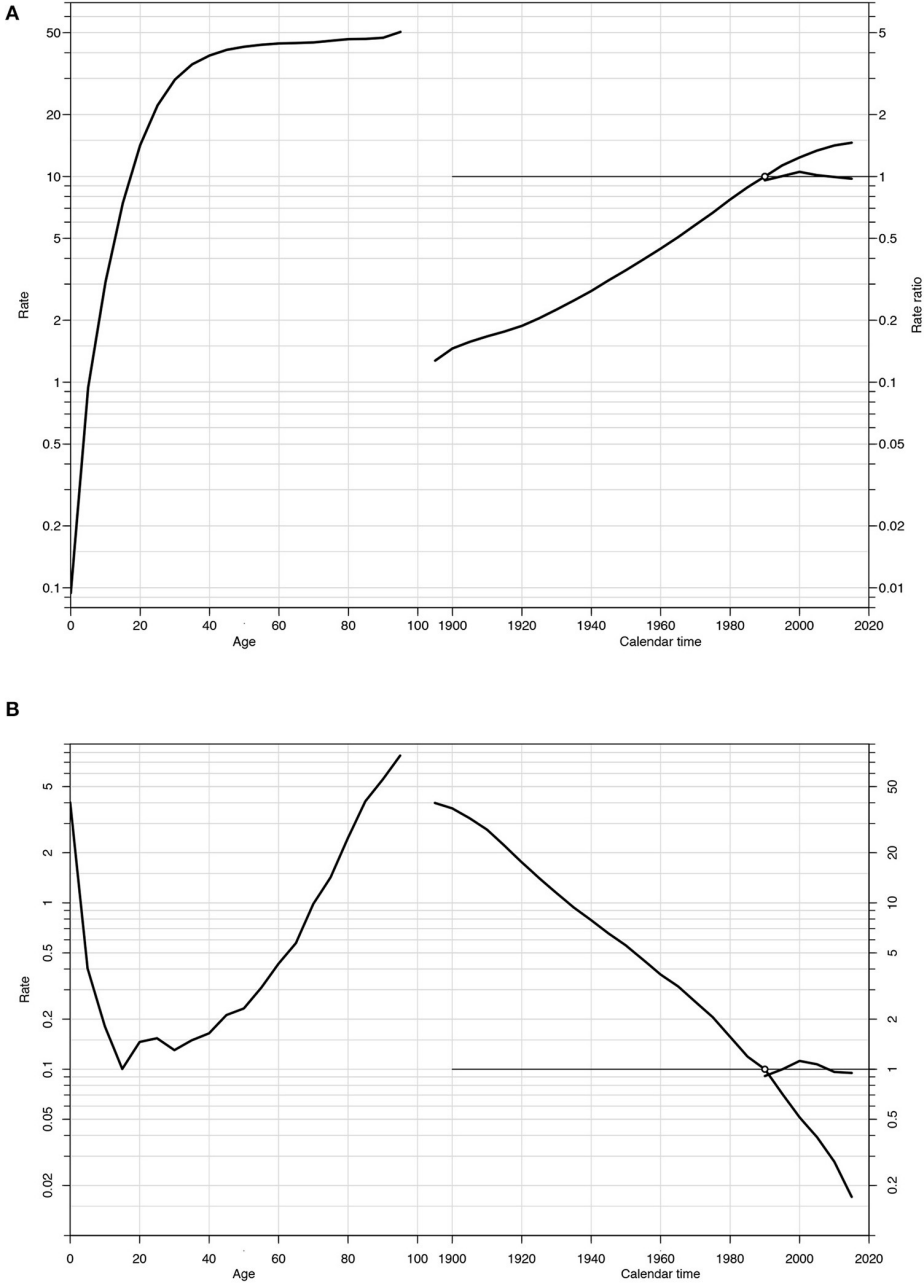
Estimated age-period-cohort effects for incidence **(A)** and mortality **(B)** of IBD in China (1990–2019). Note: Each graph has three curves depicting, from left to right, trends in the incidence or mortality rate by age for the reference cohort (age effect), incidence or mortality risk by birth cohort (cohort effect, taking 1990–1995 as the reference) and incidence or mortality risk by calendar year (period effect, taking the incidence or mortality average of the period as the reference). The graph has the horizontal axis divided into two parts: one for age (years) and one for the cohort period (calendar years). The left vertical axis represents incidence rates **(A)** or mortality rates **(B)** for the age effect. The right vertical axis represents the relative risk for the cohort and period effect. The drift is added to the non-linear birth cohort effects and the right plot presents the period effect as residual ratio rates.

The goodness of fit results for the models are summarized in Table 3. When compared to two factors only, the total model reflects the best fit of the individual effects of age, period, and cohort. Figure 7 depicts the effects of age, period, and cohort on incidence (Figure 7A and Supplementary Table 2) and mortality (Figure 7B and Supplementary Table 3) rates, allowing comparison of the slopes of the effects to be performed. The age effect for IBD incidence shows a rapidly increasing slope up to the age of 40 years, after which it flattens out. The period effect curves show no significant fluctuations. The cohort effect demonstrates a continuous increase in incidence risk from the early birth cohort to the later birth cohort. The age effect for IBD mortality decreased before the age of 15 years and increased rapidly after that age. The mortality period effect curves did not vary significantly. The mortality cohort effect, in contrast to the incidence, demonstrated a continuous decrease in risk from the early birth cohort to the later birth cohort.

**Table 3:** Comparison of age-period-cohort sub-models for the incidence and mortality of IBD.

	**Incidence**				**Mortality**				
**Model**	**AIC**	**Mod.deviance**	**Test deviance**	**Pr(>Chi)**	**AIC**	**Mod. deviance**	**Test deviance**	**Pr(>Chi)**	**H0**
Age	44499.015	43292.481	NA	NA	21831.451	20776.2048	NA	NA	
Age-drift	2915.313	1706.778	41585.7025	0.000000e+00	2534.449	1477.2030	19299.0019	0.000000e+00	Zero drift
Age-cohort	2618.683	1364.148	342.6301	1.012602e-58	2046.221	942.9750	534.2279	2.606219e-98	Coh eff | dr.
Age-period-cohort	1887.530	624.996	739.1524	1.158704e-158	1322.914	211.6685	731.3065	5.795250e-157	Per eff | Coh
Age-period	2222.930	1006.396	381.3996	1.182300e-66	1845.249	780.0032	568.3346	1.954327e-105	Coh eff | Per
Age-drift	2915.313	1706.778	700.3829	2.879636e-150	2534.449	1477.2030	697.1998	1.407857e-149	Per eff | dr.

### The burden of IBD in China compared to the global situation

**Figure 8:**
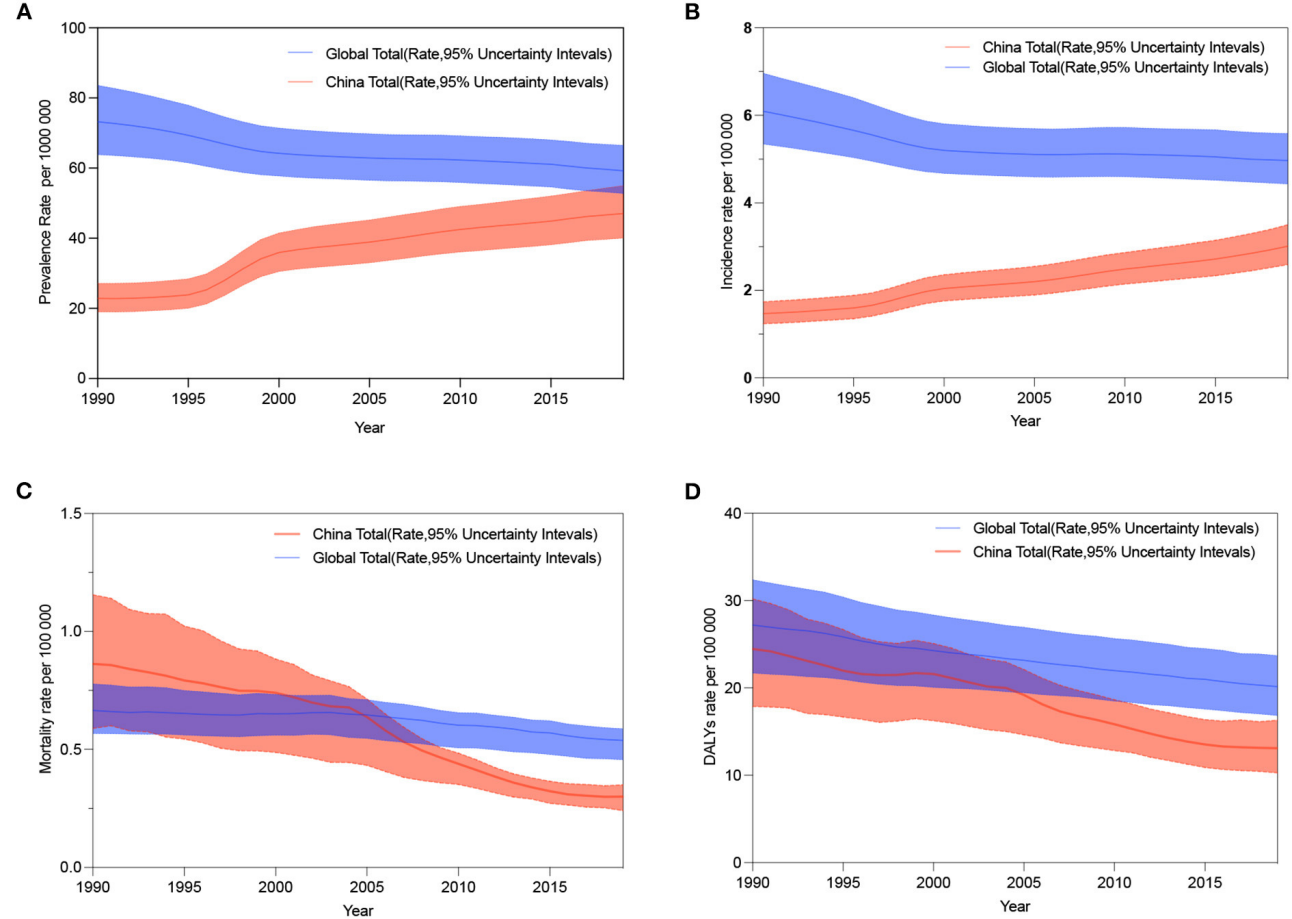
The age-standardized prevalence, incidence, mortality, and DALYs rates of IBD by both sexes from 1990 to 2019 in China compared with global values. **(A)** Trends in the age-standardized prevalence rate in China and worldwide. **(B)** Trends of age-standardized incidence rate in China and worldwide. **(C)** Trends in the age-standardized mortality rate in China and worldwide. **(D)** Trends in the age-standardized DALYs rate in China and worldwide.

Figure 8 illustrates the trends in prevalence, incidence, mortality, and DALYs rates for IBD in China and worldwide for both sexes combined from 1990 to 2019. Although the mortality rate and DALY burden in China had fallen below global levels (Figures 8C,D), the prevalence and incidence rates show opposite trends to those of the world (Figures 8A,B). It can be seen that the global prevalence and incidence of IBD are flatly declining while the same parameters for China are rapidly increasing. The burden of IBD in China remains a challenge.

## Discussion

This study examines the trends in the burden of IBD in China over the last 30 years. To our knowledge, this is the first analysis of the epidemiological trends of IBD in China using the joinpoint analysis combined with the apc model. Based on GBD 2017 data, Qiu et al. published a simple study on IBD in China (25). We discovered a substantial variation in the data after the release of GBD 2019. For example, GBD 2017 (https://gbd2017.healthdata.org/gbd-search/) published the ASIR and ASPR of IBD in China in 2017 as 60.37 (56.06, 65.36) and 136.25 (125.37, 147.44), respectively; however, GBD 2019 (https://vizhub.healthdata.org/gbd-results/) published the ASIR and ASPR as 2.85 (2.45, 3.3) and 46.20 (39.37, 53.60), respectively. As previously stated, the cause of this mismatch could be related to algorithm and model improvements. Because such a large gap can skew the understanding of IBD in China, it is vital to assess it using the most recent data release.

From 1990 to 2019, the increase in the age-standardized prevalence rates is due to the decrease in age-standardized mortality rates and DALYs, compounded by the increase in age-standardized incidence rates, which demonstrates that the burden of IBD in China has not decreased. Over the last three decades, China's economic development and social milieu have changed considerably (26). Previous studies have discovered that industrialization and urbanization predispose people to IBD (27, 28). There is substantial evidence that the prevalence of IBD in other developing Asian regions follows the same tendency observed in China (29–31).

However, current developments in medical technology have increased physicians' abilities to manage IBD. Also, the enhancement in the quality of medical care has ensured disease control and prognosis survival for IBD patients (32). The increased incidence will result in a widespread prevalence of IBD, which is not a positive omen since China is still a developing nation and has the largest population in the world. He et al. gathered hospitalization data for patients with IBD in China from the Hospital Quality Monitoring System (HQMS) database from 2013 to 2018. According to their study, hospitalization rates of IBD patients increased from 2.20 (95% CI = 2.17, 2.22) to 3.62 (3.59, 3.65) per 100,000 inhabitants, and hospitalization costs associated with IBD reached $426 million in 2018 (33). The insufficient health care coverage system and uneven medical conditions exacerbate the financial pressures and difficulties in accessing health care faced by IBD patients (34).

IBD is most commonly diagnosed in middle-aged people (35), and our findings reveal that the current peak in incidence observed in China is focused on people aged 30–45 years. IBD prevalence varies with age cohort, with 60–69 years being the peak, which is consistent with global trends (36). The joinpoint and apc analysis revealed that the incidence of IBD in China increased from 1990 to 2019, with a relative decrease in mortality. Age effects have a greater impact on IBD incidence and mortality, with its incidence being concentrated in youths and mortality risk increasing with age. In the cohort effect, the early birth cohort had a relatively low risk of incidence and a high risk of mortality. In China, the mortality rate of IBD after the age of 60 years is increasing rapidly. The high mortality rate of existing elderly IBD patients is a big problem as the Chinese population is aging. China will have 400 million inhabitants aged above 65 years by 2050 (37), which may have an effect on the IBD mortality rate. Contrary to other findings, the burden of IBD was surprisingly higher in Chinese males than in Chinese females in our study. The GBD 2017 has previously published studies demonstrating that women experience a greater worldwide burden of IBD than men do (36).

In contrast to other industrialized countries such as the United States and the United Kingdom, the incidence of IBD in the West has leveled off or even declined (38–40); however, the incidence of IBD in China is still on the rise. Although China's total incidence is now lower than the global average, if not carefully controlled, China is on track to become the country with the greatest number of IBD patients. In addition, the long-term inflammation in IBD raises the risk of cancer (41, 42). According to a single-center cohort study conducted at Peking Union Medical College Hospital, the total cancer standardized incidence ratio in IBD patients was 1.77 (95% CI: 1.33, 2.32) (43). In China, there is still a long way to go in terms of optimizing IBD management and controlling disease progression.

There are several limitations to this study. The paucity of reliable prevalence data is one of the key constraints in estimating the burden of IBD in China. There is still an unmet need for a nationwide consolidated IBD database in China (44), and estimation data based on complicated statistical models may be inaccurate. Second, GBD does not distinguish between UC and CD-related variables, making it impossible to compare their prevalence and incidence rates in the Chinese population. As a result, this study only looked at trends in the overall burden of IBD. Finally, because China has a huge geographical region with various ethnic groups, the amount of data available to assess the differences in frequency among different provinces and ethnic groups in China is limited.

The Chinese IBD Elite Union was founded in 2017 and has since come up with a number of IBD research initiatives on the Chinese population. In China, physicians are attempting to bring fresh insights into the clinical scientific difficulties surrounding IBD. We believe that when more thorough disease data will be available, further detailed epidemiological studies of IBD based on the Chinese population will become more meaningful.

## Conclusion

In conclusion, the increasing age-standardized prevalence rates are due to contributions from the reduction in age-standardized mortality rates and DALYs, compounded by the rise in the age-standardized incidence rates between 1990 and 2019 in China. IBD was more prevalent in men than in women. While the risk of mortality increased with age, the incidence was higher in young people. Although China currently has a low prevalence of IBD compared to the global level, the growing incidence has to be taken seriously. Numerous alternatives should be taken into consideration in the future to minimize the burden of IBD due to the enormous population and growing trend of aging.

## Data availability statement

The datasets presented in this study can be found in online repositories. The names of the repository/repositories and accession number(s) can be found below: All the data may be available from the IHME website (https://vizhub.healthdata.org/gbd-results/).

## Author contributions

YZ contributed to the conception of the study and wrote the first draft of the manuscript. JLiu was involved in data analysis and visualization. XH and HJ performed the data search back-to-back. LZ and JH contributed to the discussion part of the manuscript. LS and JLi reviewed and revised the manuscript. All authors contributed to the framework construction, result interpretation, and manuscript revision. All authors approved the final version of the manuscript. The corresponding authors attest that all listed authors meet authorship criteria and that no others who meet these criteria have been omitted.

## Funding

This work was supported by the Innovation One Hundred Million Talent Project Qihuang Scholar and the National Key R&D Program of China [2018YFC1705403].

## Conflict of interest

The authors declare that the research was conducted in the absence of any commercial or financial relationships that could be construed as a potential conflict of interest.

## Publisher's note

All claims expressed in this article are solely those of the authors and do not necessarily represent those of their affiliated organizations, or those of the publisher, the editors and the reviewers. Any product that may be evaluated in this article, or claim that may be made by its manufacturer, is not guaranteed or endorsed by the publisher.

## Abbreviations

IBD: inflammatory bowel disease
YLDs: years of life lived with disability
YLLs: years of life lost
DALYs: disability-adjusted life years
APC: annual percentage change
AAPC: average annual percentage change
apc: Age-period-cohort
ASPR: age-standardized rates of prevalence
ASIR: age-standardized rates of incidence
ASMR: age-standardized rates of mortality.
