# Supplementary material for: Long-term trends in the burden of inflammatory bowel disease in China over three decades: A joinpoint regression and age-period-cohort analysis based on GBD 2019
Source: Front Public Health. 2022 Sep 7;10:994619. doi: 10.3389/fpubh.2022.994619 (PMC9490087; doi:10.3389/fpubh.2022.994619)
Supplement: Supplementary file 1 [file Data_Sheet_1.PDF]

Supplementary Table 1. Annual rate of change of age-standardized rates in prevalence, incidence, deaths, YLLs, YLDs, and DALYs for IBD between 1990 and 2019 in China and Global level.

**Measure**	China			Global		
	1990	2019	Change	1990	2019	Change
Prevalence	22.85(18.98,27.15)	47.06(40.05,54.99)	1.06(0.97,1.16)	73.23(63.86,83.63)	59.25(52.78,66.47)	-0.19(-0.21,-0.17)
Incidence	1.47(1.24,1.74)	3.01(2.59,3.50)	1.04(0.96,1.14)	6.10(5.35,6.96)	4.97(4.43,5.59)	-0.18(-0.20,-0.17)
Deaths	0.86(0.59,1.16)	0.30(0.24,0.35)	-0.65(-0.76,-0.50)	0.67(0.57,0.78)	0.54(0.46,0.59)	-0.19(-0.29,-0.09)
DALYs	24.47(17.88,30.19)	13.10(10.29,16.31)	-0.46(-0.60,-0.21)	27.20(21.70,32.39)	20.15(16.86,23.71)	-0.26(-0.32,-0.17)
YLDs	3.49(2.30,4.94)	7.07(4.65,9.86)	1.03(0.92,1.15)	10.73(7.16,14.82)	8.80(5.90,12.05)	-0.18(-0.20,-0.15)
YLLs	20.98(14.62,26.46)	6.02(4.78,6.95)	-0.71(-0.78,-0.54)	16.47(12.78,19.33)	11.35(9.89,12.70)	-0.31(-0.40,-0.17)

DALYs = disability-adjusted life-years, YLDs = years lived with disability, YLLs = years of life lost.

Supplementary Table 2. Age-period cohort analysis of IBD incidence rate in China, 1990-2019.

	Male				Female			
	Estimate	Std. Error	z value	P	Estimate	Std.Error	zvalue	P
Age								
5-9	2.24956	0.07432	30.269	<2e-16***	2.162461	0.073437	29.446	<2e-16***
10-14	3.38461	0.10427	32.461	<2e-16***	3.205084	0.079354	40.390	<2e-16***
15-19	4.20633	0.14210	29.600	<2e-16***	3.972015	0.091435	43.441	<2e-16***
20-24	4.78037	0.18276	26.156	<2e-16***	4.524956	0.107092	42.253	<2e-16***
25-29	5.13657	0.22468	22.862	<2e-16***	4.881689	0.124886	39.089	<2e-16***
30-34	5.32951	0.26725	19.942	<2e-16***	5.095368	0.144007	35.383	<2e-16***

35-39	5.40275	0.31021	17.416	<2e-16***	5.194546	0.163976	31.679	<2e-16***
40-44	5.40634	0.35341	15.297	<2e-16***	5.218892	0.184522	28.283	<2e-16***
45-49	5.37730	0.39678	13.552	<2e-16***	5.205086	0.205475	25.332	<2e-16***
50-54	5.32137	0.44027	12.087	<2e-16***	5.158564	0.226724	22.753	<2e-16***
55-59	5.25596	0.48384	10.863	<2e-16***	5.096424	0.248193	20.534	<2e-16***
60-64	5.18494	0.52747	9.830	<2e-16***	5.025339	0.269824	18.625	<2e-16***
65-69	5.10659	0.57116	8.941	<2e-16***	4.943015	0.291593	16.952	<2e-16***
70-74	5.03298	0.61489	8.185	2.72e-16***	4.860316	0.313451	15.506	<2e-16***
75-79	4.97513	0.65865	7.554	4.24e-14***	4.789211	0.335409	14.279	<2e-16***
80-84	4.92205	0.70249	7.007	2.44e-12***	4.716902	0.357503	13.194	<2e-16***
85-89	4.86489	0.74670	6.515	7.26e-11***	4.637417	0.379971	12.205	<2e-16***
90-94	4.82553	0.79533	6.067	1.30e-09***	4.588864	0.403979	11.359	<2e-16***
95+	4.76936	0.80074	5.956	2.58e-09***	4.583680	0.414047	11.070	<2e-16***
Period								
1995-1999	0.11420	0.04429	2.579	0.00992**	0.163618	0.023448	6.978	2.99e-12***
2000-2004	0.22756	0.08804	2.585	0.00975**	0.332427	0.045344	7.331	2.28e-13***
2005-2009	0.27290	0.13193	2.068	0.03860*	0.388412	0.067640	5.742	9.34e-09***
2010-2014	0.33547	0.17585	1.908	0.05643.	0.459739	0.090025	5.107	3.28e-07***
2015-2019	0.39914	0.21978	1.816	0.06935.	0.531918	0.112422	4.731	2.23e-06***
Cohort								
1900-1904	-0.46954	1.06392	-0.441	0.65897	-0.117716	0.525197	-0.224	0.823
1905-1909	-0.44773	0.96458	-0.464	0.64252	-0.141726	0.477164	-0.297	0.766
1910-1914	-0.45046	0.91599	-0.492	0.62288	-0.187292	0.451485	-0.415	0.678
1915-1919	-0.46136	0.87165	-0.529	0.59660	-0.244263	0.429262	-0.569	0.569
1920-1924	-0.46857	0.82812	-0.566	0.57152	-0.290693	0.408041	-0.712	0.476
1925-1929	-0.46113	0.78487	-0.588	0.55685	-0.311184	0.387560	-0.803	0.422

1930-1934	-0.44494	0.74162	-0.600	0.54854	-0.316907	0.367056	-0.863	0.388
1935-1939	-0.42431	0.69850	-0.607	0.54355	-0.314631	0.346858	-0.907	0.364
1940-1944	-0.39906	0.65551	-0.609	0.54266	-0.304484	0.326989	-0.931	0.352
1945-1949	-0.36074	0.61265	-0.589	0.55598	-0.277042	0.307467	-0.901	0.368
1950-1954	-0.33403	0.56995	-0.586	0.55783	-0.246794	0.288372	-0.856	0.392
1955-1959	-0.30356	0.52747	-0.576	0.56495	-0.207924	0.269807	-0.771	0.441
1960-1964	-0.28108	0.48525	-0.579	0.56242	-0.158270	0.251892	-0.628	0.530
1965-1969	-0.24117	0.44336	-0.544	0.58647	-0.111716	0.234746	-0.476	0.634
1970-1974	-0.19777	0.40192	-0.492	0.62268	-0.053067	0.218580	-0.243	0.808
1975-1979	-0.15043	0.36107	-0.417	0.67696	0.001151	0.203623	0.006	0.995
1980-1984	-0.08833	0.32105	-0.275	0.78323	0.063446	0.190154	0.334	0.739
1985-1989	-0.02829	0.28219	-0.100	0.92013	0.098181	0.178491	0.550	0.582
1990-1994	0.02063	0.24505	0.084	0.93292	0.112374	0.169009	0.665	0.506
1995-1999	0.06072	0.21060	0.288	0.77310	0.135417	0.162213	0.835	0.404
2000-2004	0.06839	0.18043	0.379	0.70468	0.124776	0.158531	0.787	0.431
2005-2009	0.06533	0.15737	0.415	0.67802	0.103359	0.158666	0.651	0.515
2010-2014	0.04525	0.14687	0.308	0.75802	0.063495	0.165115	0.385	0.701
2015-2019	NA	NA	NA	NA	NA	NA	NA	NA
Residual deviance	544.33				151.75			
AIC	1723.2				1323.4			

Significance codes: ***<0.001 **<0.01 *<0.05 .<0.1

Supplementary Table 3. Age-period cohort analysis of IBD mortality rate in China, 1990-2019.

	Male				Female			
	Estimate	Std.Error	zvalue	Pr(>|z|)	Estimate	Std.Error	zvalue	Pr(>|z|)
Age								
5-9	-1.685471	0.038889	-43.340	<2e-16***	-2.45482	0.04516	-54.359	<2e-16***
10-14	-2.397957	0.053057	-45.196	<2e-16***	-2.85039	0.05301	-53.769	<2e-16***
15-19	-2.756394	0.063689	-43.279	<2e-16***	-3.21333	0.06144	-52.304	<2e-16***
20-24	-1.906055	0.056486	-33.744	<2e-16***	-2.90729	0.05823	-49.931	<2e-16***
25-29	-1.666291	0.061144	-27.252	<2e-16***	-2.56991	0.05694	-45.132	<2e-16***
30-34	-1.574340	0.068342	-23.036	<2e-16***	-2.55290	0.06104	-41.820	<2e-16***
35-39	-1.291526	0.073964	-17.462	<2e-16***	-2.07710	0.05991	-34.669	<2e-16***
40-44	-0.920729	0.079731	-11.548	<2e-16***	-1.81651	0.06084	-29.858	<2e-16***
45-49	-0.359398	0.085743	-4.192	2.77e-05***	-1.46243	0.06179	-23.667	<2e-16***
50-54	-0.078918	0.092895	-0.850	0.39558	-1.08330	0.06314	-17.156	<2e-16***
55-59	0.467831	0.099904	4.683	2.83e-06***	-0.62333	0.06461	-9.648	<2e-16***
60-64	0.981281	0.107161	9.157	<2e-16***	0.01517	0.06586	0.230	0.817830
65-69	1.462423	0.114749	12.744	<2e-16***	0.57970	0.06795	8.532	<2e-16***
70-74	2.222897	0.122391	18.162	<2e-16***	1.37068	0.07024	19.515	<2e-16***
75-79	2.805703	0.130307	21.531	<2e-16***	2.00333	0.07311	27.402	<2e-16***
80-84	3.595625	0.138318	25.995	<2e-16***	2.79087	0.07624	36.608	<2e-16***
85-89	4.523883	0.146505	30.879	<2e-16***	3.43769	0.07981	43.073	<2e-16***
90-94	5.115066	0.155951	32.799	<2e-16***	4.06895	0.08399	48.448	<2e-16***
95+	5.269325	0.153258	34.382	<2e-16***	4.75320	0.08462	56.172	<2e-16***
Period								

1995-1999	-0.144208	0.015284	-9.435	<2e-16***	-0.14485	0.01306	-11.087	<2e-16***
2000-2004	-0.271464	0.021235	-12.784	<2e-16***	-0.25733	0.01538	-16.726	<2e-16***
2005-2009	-0.497882	0.028714	-17.339	<2e-16***	-0.58815	0.01920	-30.637	<2e-16***
2010-2014	-0.748282	0.036748	-20.363	<2e-16***	-1.02020	0.02367	-43.095	<2e-16***
2015-2019	-0.960469	0.044686	-21.494	<2e-16***	-1.30249	0.02738	-47.567	<2e-16***
Cohort								
1900-1904	-0.166682	0.197344	-0.845	0.39832	0.22872	0.08821	2.593	0.009516**
1905-1909	-0.094594	0.181476	-0.521	0.60219	0.37179	0.08058	4.614	3.96e-06***
1910-1914	-0.015796	0.172661	-0.091	0.92711	0.46473	0.07677	6.054	1.42e-09***
1915-1919	-0.003066	0.164583	-0.019	0.98514	0.49579	0.07387	6.712	1.92e-11***
1920-1924	0.012656	0.156761	0.081	0.93565	0.50902	0.07131	7.138	9.45e-13***
1925-1929	0.032250	0.149195	0.216	0.82886	0.52700	0.06949	7.584	3.36e-14***
1930-1934	0.053763	0.141588	0.380	0.70416	0.55065	0.06778	8.124	4.50e-16***
1935-1939	0.086965	0.134269	0.648	0.51718	0.57872	0.06660	8.689	<2e-16***
1940-1944	0.154313	0.127236	1.213	0.22520	0.61360	0.06616	9.275	<2e-16***
1945-1949	0.203377	0.120400	1.689	0.09118.	0.65272	0.06607	9.880	<2e-16***
1950-1954	0.261609	0.113812	2.299	0.02153*	0.71992	0.06617	10.880	<2e-16***
1955-1959	0.285924	0.107568	2.658	0.00786**	0.74634	0.06675	11.181	<2e-16***
1960-1964	0.316737	0.102028	3.104	0.00191**	0.75367	0.06868	10.974	<2e-16***
1965-1969	0.381172	0.096032	3.969	7.21e-05***	0.81793	0.06843	11.953	<2e-16***
1970-1974	0.396827	0.090870	4.367	1.26e-05***	0.82673	0.06980	11.845	<2e-16***
1975-1979	0.426614	0.087606	4.870	1.12e-06***	0.82533	0.07261	11.367	<2e-16***
1980-1984	0.434471	0.084745	5.127	2.95e-07***	0.70614	0.07562	9.338	<2e-16***
1985-1989	0.381640	0.078780	4.844	1.27e-06***	0.67556	0.07332	9.214	<2e-16***
1990-1994	0.378929	0.066644	5.686	1.30e-08***	0.75855	0.05957	12.734	<2e-16***
1995-1999	0.335024	0.066084	5.070	3.99e-07***	0.61778	0.06304	9.800	<2e-16***

2000-2004	0.343549	0.066759	5.146	2.66e-07***	0.44668	0.06738	6.630	3.36e-11***
2005-2009	0.347962	0.069192	5.029	4.93e-07***	0.36295	0.07370	4.925	8.44e-07***
2010-2014	0.220239	0.075021	2.936	0.00333**	0.27931	0.08273	3.376	0.000735***
2015-2019	NA	NA	NA	NA	NA	NA	NA	NA
Residual deviance	88.657				197.65			
AIC	1115.2				1215.6			

Significance codes: ***<0.001 **<0.01 *<0.05 .<0.1
