# Supplementary material for: Long-term trends in the burden of inflammatory bowel disease in China over three decades: A joinpoint regression and age-period-cohort analysis based on GBD 2019
Source: Front Public Health. 2022 Sep 7;10:994619. doi: 10.3389/fpubh.2022.994619 (PMC9490087; doi:10.3389/fpubh.2022.994619)
Supplement: Supplementary file 2 [file Data_Sheet_2.PDF]

Supplementary Figures

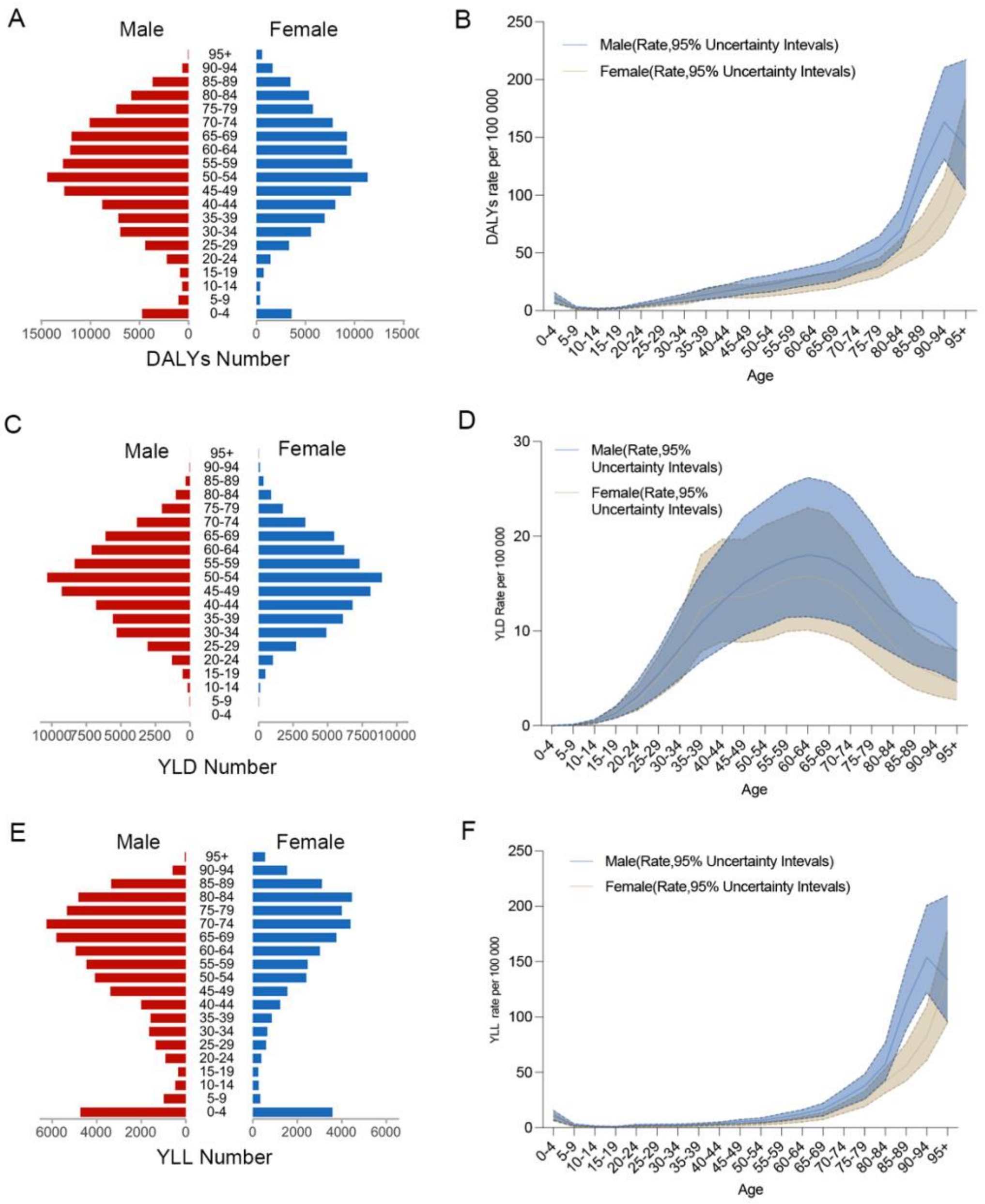

Supplementary Figure 1. Age-specific numbers and age-standardized DALYs, YLD, YLL rates of IBD in China, 2019. (**A**) Age-specific DALYs number. (**B**) Age-standardized DALYs rate. (**C**) Age-specific YLD number. (**D**) Age-standardized YLD rate. (**E**) Age-specific YLL number. (**F**) Age-standardized YLL rate.

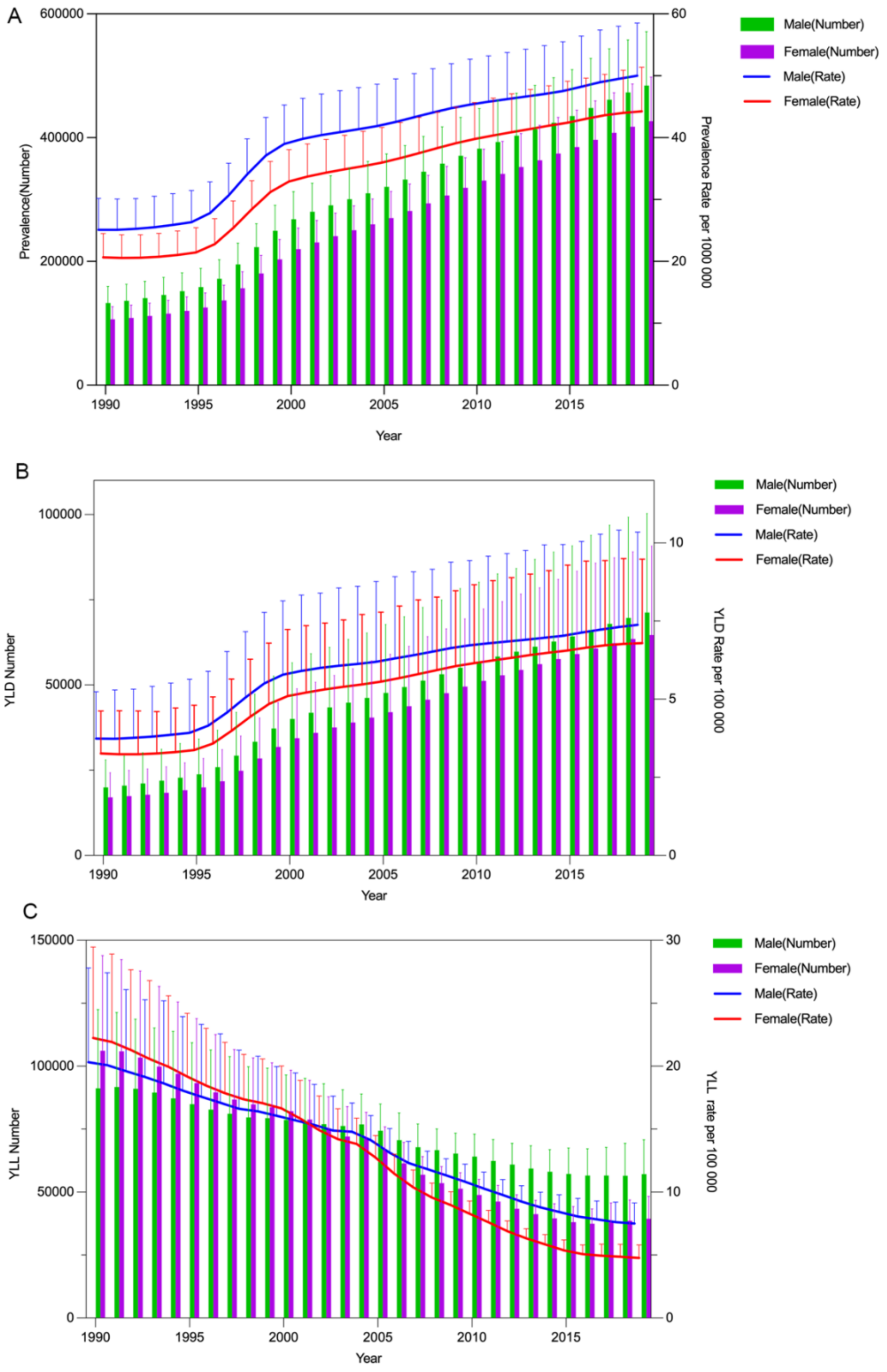

Supplementary Figure 2. Trends in the all-age cases and age-standardized prevalence, YLD, YLL rates of IBD by sex from 1990 to 2019. (**A**) Prevalence number and rate. (**B**)YLD number and rate. (**C**) YLL number and rate.

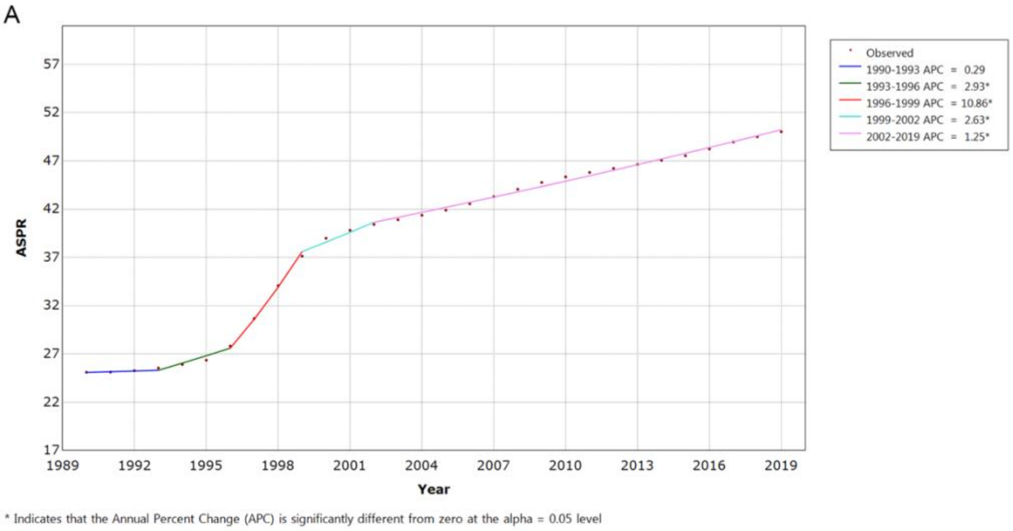

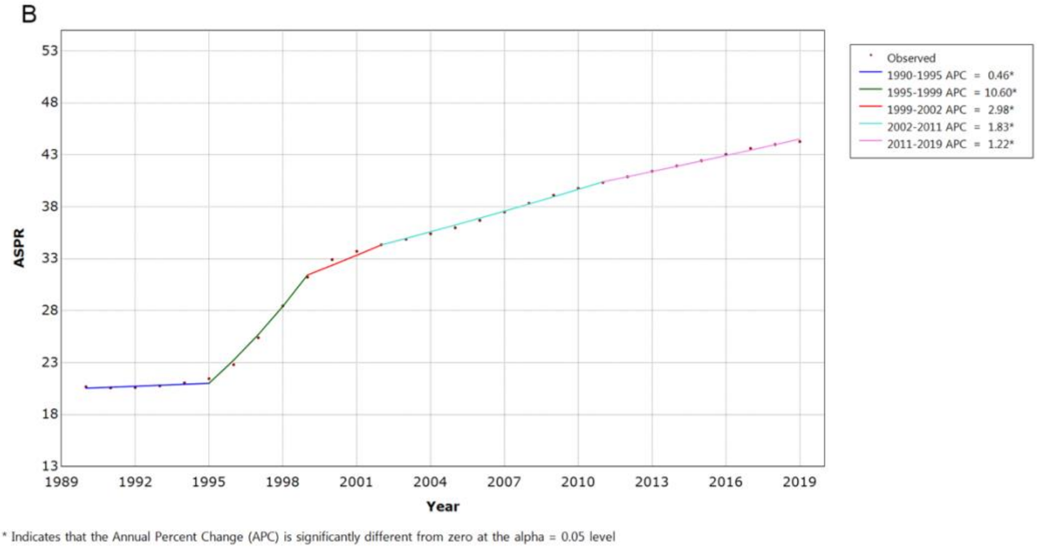

Supplementary Figure 3. Joinpoint regression analysis of the sex-specific age-standardized prevalence rate for IBD in China from 1990 to 2019. (**A**) Age-standardized prevalence rate for males. (**B**) Age-standardized prevalence rate for females.

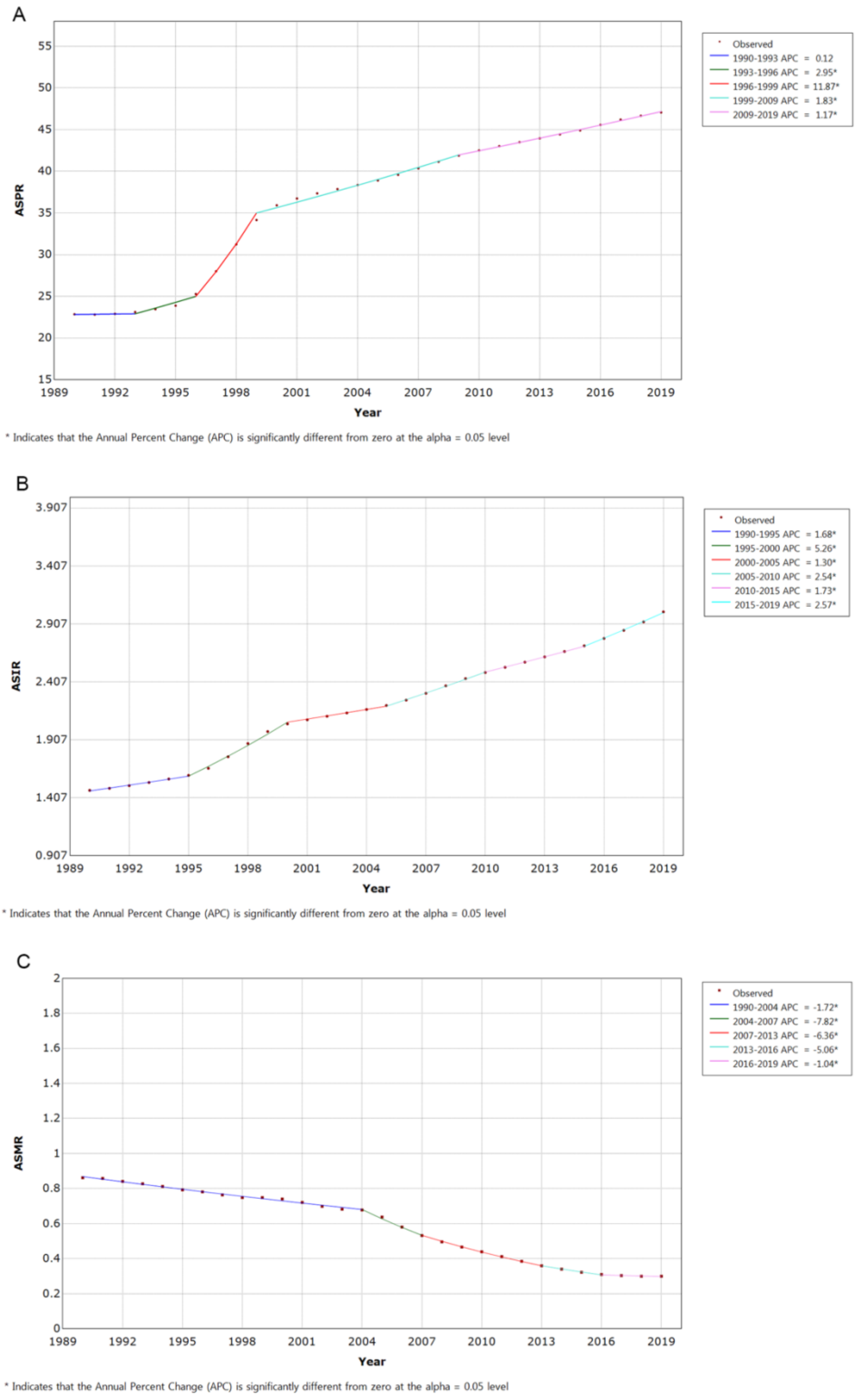

Supplementary Figure 4. Joinpoint regression analysis of the both sex age-standardized prevalence, incidence, and mortality rate for IBD in China from 1990 to 2019. (**A**) Age-standardized prevalence rate. (**B**) Age-standardized incidence rate. (**C**) Age-standardized mortality rate.

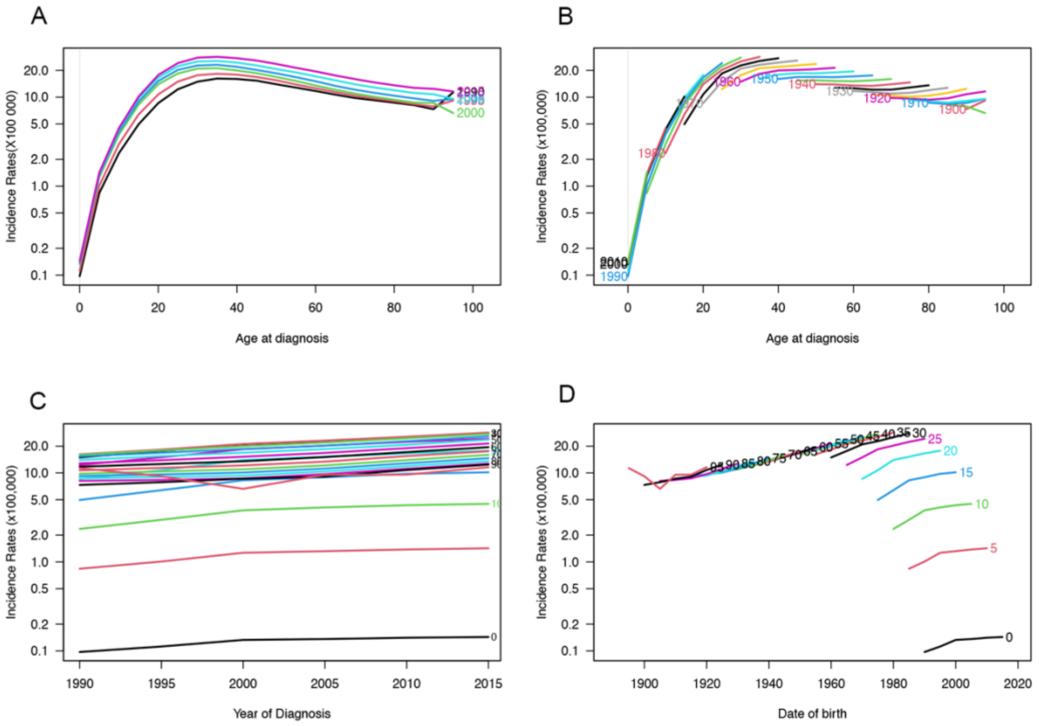

Supplementary Figure 5. Incidence rates of IBD in China among males. (**A**) The age-specific incidences rates of IBD according to time period; each line connects the age-specific incidence for a 5-year period. (**B**) The age-specific incidences rates of IBD according to birth cohort; each line connects the age-specific incidence for a 5-year cohort. (**C**)The period–specific incidence rates of IBD according to age group; each line connects the birth cohort–specific incidence for a 5-year age group. (**D**)The birth cohort–specific incidence rates of IBD according to age group; each line connects the birth cohort–specific incidence for a 5-year age group.

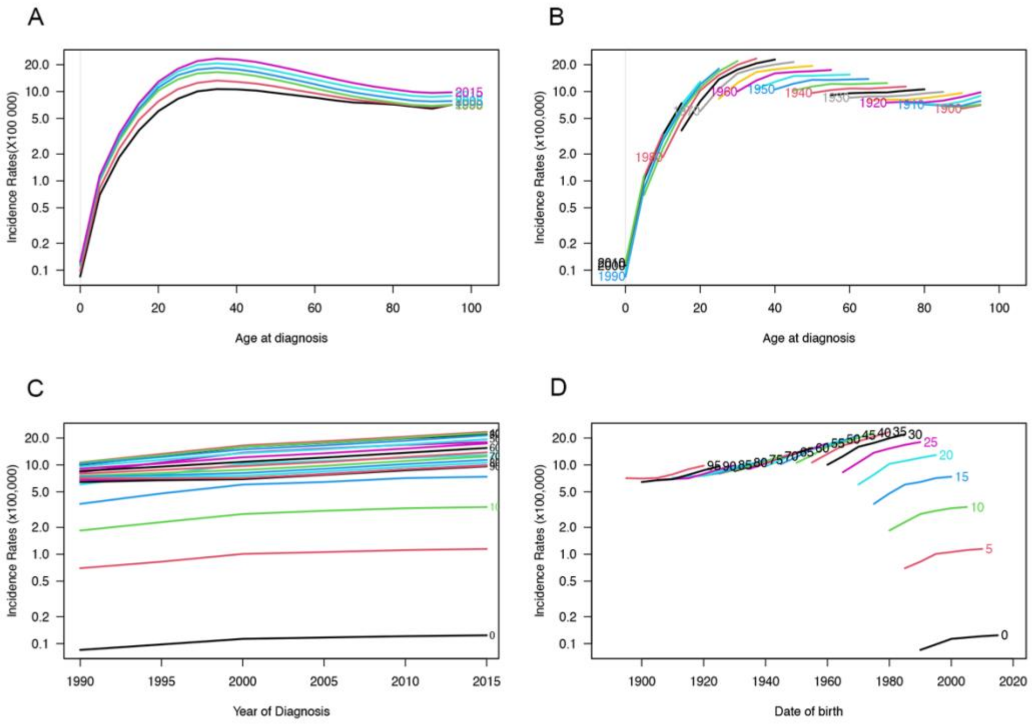

Supplementary Figure 6. Incidence rates of IBD in China among females. (**A**) The age-specific incidences rates of IBD according to time period; each line connects the age-specific incidence for a 5-year period. (**B**) The age-specific incidences rates of IBD according to birth cohort; each line connects the age-specific incidence for a 5-year cohort. (**C**)The period–specific incidence rates of IBD according to age group; each line connects the birth cohort–specific incidence for a 5-year age group. (**D**)The birth cohort–specific incidence rates of IBD according to age group; each line connects the birth cohort–specific incidence for a 5-year age group.

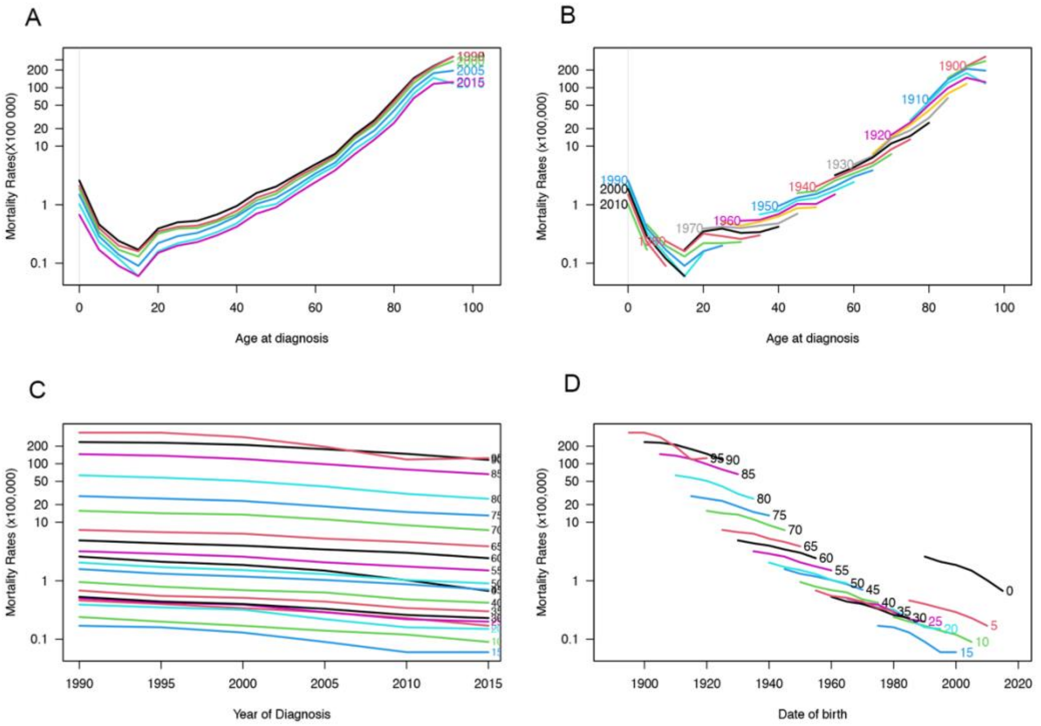

Supplementary Figure 7. Mortality rates of IBD in China among males. (**A**) The age-specific incidences rates of IBD according to time period; each line connects the age-specific incidence for a 5-year period. (**B**) The age-specific incidences rates of IBD according to birth cohort; each line connects the age-specific incidence for a 5-year cohort. (**C**)The period–specific incidence rates of IBD according to age group; each line connects the birth cohort–specific incidence for a 5-year age group. (**D**)The birth cohort–specific incidence rates of IBD according to age group; each line connects the birth cohort–specific incidence for a 5-year age group.

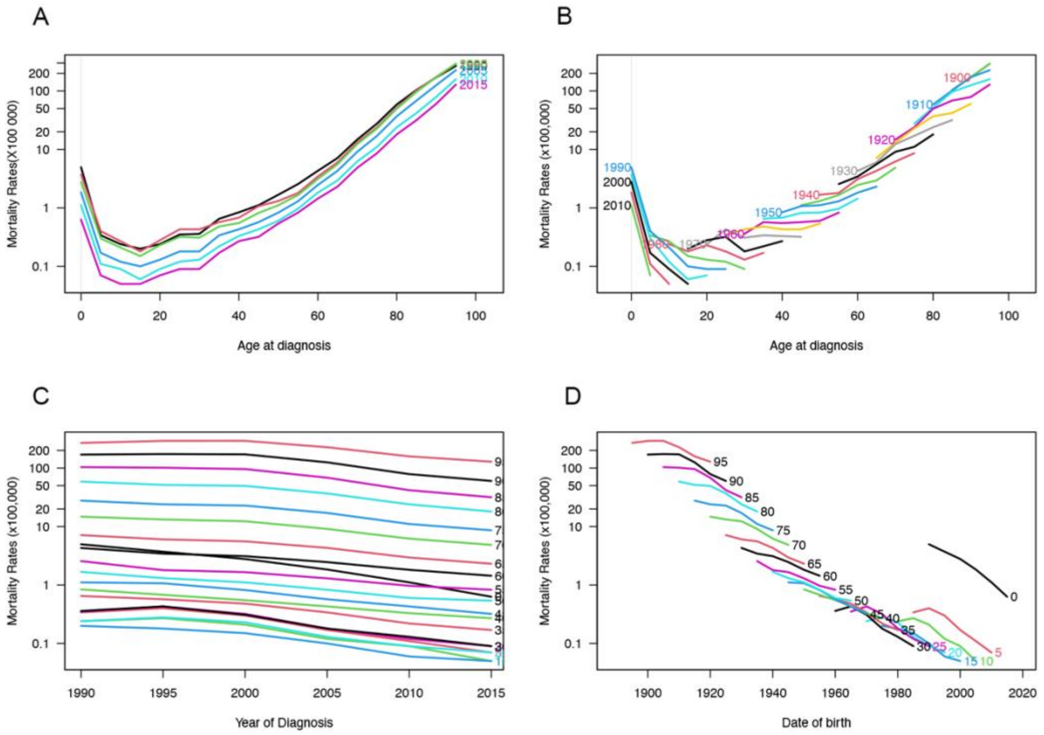

Supplementary Figure 8. Mortality rates of IBD in China among females. (**A**) The age-specific incidences rates of IBD according to time period; each line connects the age-specific incidence for a 5-year period. (**B**) The age-specific incidences rates of IBD according to birth cohort; each line connects the age-specific incidence for a 5-year cohort. (**C**)The period–specific incidence rates of IBD according to age group; each line connects the birth cohort–specific incidence for a 5-year age group. (**D**)The birth cohort–specific incidence rates of IBD according to age group; each line connects the birth cohort–specific incidence for a 5-year age group.
